# Supplementary material for: Genome-Wide SSR Markers Reveal Genetic Diversity and Establish a Core Collection for Commercial Hypsizygus marmoreus Germplasm
Source: J Fungi (Basel). 2025 Nov 28;11(12):842. doi: 10.3390/jof11120842 (PMC12734334; doi:10.3390/jof11120842)
Supplement: Supplementary file 1 [file jof-11-00842-s001.zip › jof-3969010-supplementary.pdf]

Journal of Fungi

Supplementary Material

Table S1: Accessions and origins of *H. marmoreus* varieties.

Strains	Origin
Finc-B-3	Jiangsu, China
H23	Jiangsu, China
HH2	Jiangsu, China
HH8	Jiangsu, China
HM15	Jiangsu, China
HM18	Jiangsu, China
HM21	Jiangsu, China
HM22	Jiangsu, China
HM23	Jiangsu, China
HM30	Jiangsu, China
HM31	Jiangsu, China
HM34	Jiangsu, China
HM35	Jiangsu, China
HM55	Jiangsu, China
HM56	Jiangsu, China
HM44	Malaysia
HM47	Malaysia
HM48	Malaysia
HM49	Malaysia
HM51	Malaysia
HM53	Malaysia
HZ22	Malaysia
HZ23	Malaysia
HZ27	Malaysia
HZ29	Malaysia
Finc-B-6	Japan
Finc-F-4	Japan
Finc-N-11	Japan
HM40	Japan
HZ1157	Japan
HZ12	Japan
HZ17	Japan
HZ18	Japan
HZ20	Japan
Finc-B-5	Shanghai, China
Finc-B-7	Shanghai, China
Finc-W-247	Shanghai, China
HM1	Shanghai, China

HM2	Shanghai, China
HM3	Shanghai, China
HM4	Shanghai, China
HM5	Shanghai, China
HM6	Shanghai, China
HM7	Shanghai, China
HM8	Shanghai, China
HM9	Shanghai, China
HM16	Tianjin, China
HM57	Tianjin, China
HM11	Shanghai, China
HM12	Shanghai, China
HM13	Shanghai, China
HM20	Shanghai, China
HM24	Shanghai, China
HM37	Shanghai, China
HM38	Shanghai, China
HM54	Shanghai, China
HZ6	Shanghai, China

.

Table S2: Supplementary coding information of *H. marmoreus* germplasm

Complement code	Complement code information		
Specific genetic information	T: Transgenic	S: Molecular marker auxiliary selection	M: Mutagenic breeding
	H: Hybrid breeding	U: Unknown	
Color	B: Brown	W: White	
Origin	C: Cultivate	W: Wild	

Table S3. Quantitative trait distribution of 24 core collection of *H. marmoreus.*

Strains	Quantitative trait					
	Cap diameter (mm)	Cap thickness (mm)	Stipe length (mm)	Stipe diameter (mm)	Yield (g)	Number of fruiting body
HM13	/	/	/	/	/	/
HM37	19.49±5.23	5.94±1.22	53.08±2.63	9.2±2.63	138.59±16.67	35.93±6.81
HM6	11.63±2.91	5.38±1.1	42.08±9.76	8.5±2.61	57.86±27.89	36.64±11.57
Finc-B-3	14.25±2.07	7.41±1.36	48.67±5.1	7.01±1.07	227.77±11.67	110.36±25.07
HZ27	16.09±3.46	7.58±1.76	48.4±7.23	22.92±1.2	96.97±17.83	24.23±5.14
Finc-B-5	16.43±0.9	6.74±0.67	85.69±3.48	11.56±2.69	183.67±24.87	63.98±1.99
HM21	12.64±2.51	6.54±1.11	47.53±8.03	7.81±1.79	172.55±27.06	111.57±19.79
HZ29	15.17±2.06	6.64±1.38	60.4±4.38	8.45±1.68	153.3±18.48	43.74±8
HM22	11±2.45	5.98±1.43	36.7±4.44	7.42±2.44	93.83±18.37	108.87±13.21
HZ22	17.49±3.82	7.58±1.41	91.71±11.77	10.5±7.62	111.97±11.67	25.8±6.58
HH2	12.21±2.32	6.95±1.57	84.05±13.04	7.61±1.43	241.28±9.74	106.5±14.12
HM3	16.6±4.81	5.66±1.72	47.25±9.99	7.72±2.35	88.65±15.91	31.33±3.98
Finc-F-4	19.46±3.3	4.37±1.07	84.93±2.14	11.89±1.8	170.83±7.8	53±7.02
HM55	12.69±2.3	6.52±1.49	65.57±9.47	8.41±1.55	227.43±14.56	108.5±15.87
HZ18	14.42±2.9	7.61±1.63	75.27±10.13	7.43±1.9	190.33±17.22	86.14±16.14
HZ23	16.29±2.55	7.33±1.34	56.77±5.66	6.29±0.81	158.42±8.64	73.48±14.89
Finc-B-6	14.52±1.25	7.34±1.8	67.73±1.77	6.25±1.49	179.32±7.17	69.54±5.43
HZ11	10.62±1.81	5.99±1.09	56.33±5.17	5.73±1.26	176.07±26.43	97.39±19.13
HM31	11.06±1.61	6.67±0.97	53.14±5.44	6.07±1.12	166.35±21.93	104.27±13.67
HM30	10.15±1.39	6.31±1.11	54.78±6.37	6.31±1.18	178.36±12.6	109.67±12.44
HM35	10.71±1.88	6.35±1.08	53.86±5.76	6.66±1.32	169.96±13.5	114.52±15.09
Finc-B-7	22.35±3.67	6.89±1.55	94.56±3.87	12.67±1.61	137.58±17.25	46.92±1.92
Finc-N-11	14.89±2.02	5.84±0.69	75.94±2.58	10.78±1.94	149.67±29.61	63.09±3.82
HM18	11.1±1.66	6.08±0.83	57.36±5.09	6.3±1.14	155.47±12.83	111.35±20

Table S4. Qualitative trait distribution of 24 core collection of *H. marmoreus.*

Strains	Qualitative trait					
	Cap shape	Color of cap center	Color of cap margin	Color of gills	Arrangement of gills	Color of stipe
HM13	/	/	/	/	/	/
HM37	Flat	brown	Brown	Grayish yellow	Ripple	Yellowish white
HM6	Flat	Dark brown	Brown	Grayish yellow	Ripple	Yellowish white
Finc-B-3	Round	White	White	White	Ripple	White
HZ27	Round	White	White	White	Ripple	White
Finc-B-5	Round	Brown	Grayish white	Grayish yellow	Straight	Yellowish white
HM21	Round	White	White	White	Ripple	White
HZ29	Round	White	White	White	Ripple	White
HM22	Round	Dark brown	Brown	Grayish yellow	Straight	Yellowish white
HZ22	Round	White	White	White	Straight	White
HH2	Round	White	White	White	Ripple	White
HM3	Flat	Dark brown	Brown	Grayish yellow	Ripple	Yellowish white
Finc-F-4	Flat	Light brown	Light yellow	Grayish white	Ripple	Yellowish white
HM55	Round	Brown	Brown	Grayish yellow	Ripple	Yellowish white
HZ18	Round	Light brown	Light brown	Grayish yellow	Ripple	Yellowish white
HZ23	Round	Brown	Brown	Grayish yellow	Straight	Yellowish white
Finc-B-6	Round	Dark brown	Brown	Grayish yellow	Straight	Yellowish white
HZ11	Round	Dark brown	Brown	Grayish yellow	Ripple	Yellowish white
HM31	Round	Dark brown	Brown	Grayish yellow	Ripple	Yellowish white

HM30	Round	Dark brown	Brown	Grayish yellow	Ripple	Yellowish white
HM35	Round	Dark brown	Brown	Grayish yellow	Ripple	Yellowish white
Finc-B-7	Round	Dark brown	Brown	White	Straight	White
Finc-N-11	Round	Brown	Light brown	White	Straight	Yellowish white
HM18	Round	Light brown	Light brown	Grayish yellow	Ripple	Yellowish white
